# Detecting Uncoded Self-Harm in Veterans’ Electronic Health Records Using Positive and Unlabeled Learning: Retrospective Cohort Study

**DOI:** 10.2196/89071

**Published:** 2026-06-04

**Authors:** Praveen Kumar, Alexandria D Viszolay, Rajesh Upadhayaya, Fariha Moomtaheen, Donald R Greer, Cristian G Bologa, Kristan A Schneider, Sharon E Davis, Michael E Matheny, David van der Goes, Gerardo Villarreal, Yiliang Zhu, Mauricio Tohen, Scott A Malec, Jeremy J Yang, Elliot M Fielstein, Christophe Gerard Lambert

**Affiliations:** 1 Department of Internal Medicine, School of Medicine, University of New Mexico Health Sciences Center, 1 University of New Mexico, MSC10 5550, Albuquerque, NM, 87131, United States, 1 505-272-9709; 2 Greer Black Company, Bozeman, MT, United States; 3 Raymond G. Murphy VA Medical Center, Albuquerque, NM, United States; 4 Department of Biomedical Informatics, Vanderbilt University Medical Center, Nashville, TN, United States; 5 VA Tennessee Valley Healthcare System, Nashville, TN, United States; 6 Department of Economics, University of New Mexico, Albuquerque, NM, United States; 7 Department of Psychiatry and Behavioral Sciences, School of Medicine, University of New Mexico Health Sciences Center, Albuquerque, NM, United States; 8 Office of Mental Health, United States Department of Veterans Affairs, Washington, DC, United States

**Keywords:** self-injurious behavior, machine learning, Veterans' health, PULSNAR, electronic health record, positive unlabeled learning selected not at random

## Abstract

**Background:**

Underdiagnosis and undercoding are common across mental health conditions, particularly suicide and self-harm. This leaves health care datasets lacking reliable negative examples needed for predictive modeling, phenotype prevalence estimation, and identification of individuals at elevated risk. We use positive and unlabeled (PU) learning to address this challenge.

**Objective:**

This study aims to identify US Veterans whose self-harm events were not explicitly captured through diagnostic codes in electronic health records (EHRs) and estimate the underlying prevalence using a novel PU learning algorithm.

**Methods:**

We performed a retrospective cohort study using Veterans Health Administration EHRs (from October 1, 1999, to August 31, 2019), selecting a random 25% sample of 1,329,120 Veterans out of 5,316,480 (1,193,563 males and 135,557 females) with at least 2 years of observation. The study cohort comprised 24,625 Veterans with coded self-harm and 1,304,495 uncoded, with the mean ages of 38.39 (SD 12.17) and 48.76 (SD 15.04) years, respectively. We applied our PULSNAR (positive unlabeled learning selected not at random) algorithm to estimate the proportion of individuals with uncoded self-harm. Covariates included age, medical conditions, procedures, and clinical observations. Four experts (raters) independently reviewed charts of 97 uncoded Veterans, each selected from 1% intervals of calibrated PULSNAR probabilities from 0.01 to 0.97. Agreement was assessed among raters, PULSNAR classifications, and consensus review decisions. Post hoc calibration was used to refine prevalence estimates.

**Results:**

Of the 159,049 covariates in the dataset, PULSNAR’s Extreme Gradient Boosting (XGBoost) model identified 1302 (0.82%) as informative for classification. Only 1.85% (24,625/1,329,120) of Veterans had diagnostic codes indicating self-harm events, while PULSNAR estimated an overall prevalence of 10.46% (139,026/1,329,120) by identifying an additional *α*=8.77% (114,404/1,304,495) of self-harm cases among the uncoded population. Of the 97 chart-reviewed patients, 39 had documented but uncoded self-harm. PULSNAR probabilities were post hoc calibrated such that their sum over the 97 cases equaled 39, which adjusted the combined coded and imputed prevalence downward from 10.46% to 7.91% (105,133/1,329,120). By applying this calibration to shift the probabilities of all uncoded Veterans, with bootstrapping for confidence intervals, PULSNAR estimates that coded self-harm represents only 23.4% (95% CI 17.76% to 31.51%) of all documented (coded+notes) self-harm.

**Conclusions:**

Under the “selected not at random” assumption, PULSNAR provides an innovative and scalable framework for estimating the clinically documented prevalence of mental health conditions and identifying the uncoded individuals with calibrated prediction, without requiring confirmed negative labels. This method offers an alternative to time-consuming chart reviews for detecting likely cases missing structured coding capture. By addressing diagnostic undercoding of mental health conditions in EHRs, this approach has the potential to enhance the estimation of mental health prevalence and support screening, activation of automated clinical decision support, targeted intervention, better resource allocation, and research to improve outcomes in real-world settings.

## Introduction

Suicide and self-harm remain significant public health concerns in the United States, consistently ranking among the leading causes of death. In 2023, suicide was among the top 8 causes of death for individuals aged 10‐64 years and the second leading cause for those aged 10-34 years [1,2]. According to the Centers for Disease Control and Prevention, suicide accounted for 49,316 deaths in 2023 [1], 1.6% of all deaths. The prevalence of suicidal thoughts and attempts is even higher than suicide deaths. In 2023, an estimated 12.8 million American adults aged 18 years or older experienced serious thoughts of suicide, 3.7 million made a suicide plan, and 1.5 million attempted suicide [1,3]. These data suggest that for each suicide death, there are hundreds of individuals experiencing suicidal ideation or engaging in self-harm.

Veterans are at a disproportionately higher risk of experiencing trauma and self-destructive behavior compared to the general population, accounting for nearly 14% of adult suicide deaths despite representing only 7.6% of the population [4]. The overall unadjusted suicide rate among Veterans was roughly double that of non-Veteran adults (34.7 per 100,000 vs 17.1 per 100,000) [5]. Both male (37.3 vs 28.7 per 100,000) and female Veterans (13.5 vs 7.2 per 100,000) had markedly higher suicide rates than their non-Veteran counterparts. In addition, younger Veterans (ages 18‐34 years) faced the highest risk (47.6 per 100,000)—far exceeding that of any other age group [5]. Co-occurring conditions, such as posttraumatic stress disorder, depression, bipolar disorder, substance use disorders, traumatic brain injury, and prior self-injurious behavior, significantly contribute to this elevated risk [4,6].

Suicide rarely occurs in isolation; it is often preceded by identifiable risk factors, with trauma, past self-harm, and suicidal ideation being among the strongest predictors of future suicide risk [7,8]. Early identification of individuals exhibiting these behaviors is critical, as timely intervention can significantly reduce the risk of suicide. Studying self-harm, therefore, is essential for comprehensive suicide prevention efforts. Within the Veterans Health Administration (VHA), policy and quality metrics primarily focus on suicidal self-directed violence within the past 12 months, rather than self-harm broadly defined. Although our study centers on ever self-harm as a phenotype, it is important to distinguish this analytic focus from operational VHA surveillance, which prioritizes identification and follow-up of suicidal behaviors in the near-term versus capturing all self-harm behaviors with and without intent.

The widespread adoption of electronic health records (EHRs) in the United States has generated large repositories of patient health care data, comprising both structured data (eg, demographics, diagnoses, procedures, and prescriptions) and unstructured data (eg, clinical notes, imaging, and pathology reports) [9-11]. While these data are invaluable for observational studies and health care analytics, they also reveal inconsistencies in data quality across different clinical settings and heterogeneous data structures that span structured codes, free-text notes, and temporal measurements [11]. Additionally, these data are affected by missingness due to multiple mechanisms, including the incomplete capture of out-of-network care, underreporting of sensitive conditions, and not documenting or undercoding of clinical diagnoses. Undercoding refers to instances in which events or conditions, such as self-harm behaviors and mental illnesses, are not recorded in structured diagnosis codes in the EHR, leading to physicians overlooking past diagnoses, inaccurate prevalence estimates, and hindering effective intervention strategies. Importantly, undercoding of mental health conditions, including suicidality and self-harm, is common in EHRs, limiting research that relies on accurate reporting of these conditions [12-15]. VHA’s suicide risk surveillance relies heavily on policy-driven, standardized national note templates such as the Suicide Behavior and Overdose Report (SBOR), which capture suicidal and other self-directed violence events in structured clinical documentation. These templates function in parallel to diagnostic coding and are a primary mechanism by which VHA monitors suicide behaviors. Undercoding of *ICD* (*International Classification of Diseases*) self-harm diagnoses reflects partially the extent of VHA suicide risk monitoring efforts.

The increased availability of EHRs and advancements in machine learning (ML) methodologies have led to increased application of ML techniques to identify and predict instances of self-harm and suicidal ideation using EHRs and insurance claims data [12,13,15-22]. Kumar et al [12] and Nestsiarovich et al [13] used an Extreme Gradient Boosting (XGBoost) [23] model on visit-level data to estimate the uncoded self-harm events among individuals with major mental illness. Simon et al [16] developed random forest–based models aimed at predicting fatal or nonfatal self-harm events within 90 days following a sampled encounter. Simon et al [17] used LASSO (least absolute shrinkage and selection operator)–based logistic regression models to predict suicide attempts and suicide deaths post-outpatient visits. Rozova et al [18] used natural language processing (NLP) supervised learning techniques on free-text triage notes from emergency department (ED) visits to detect self-harm and suicidal ideation among ED patients. Walsh et al [19] used random forest and nonregularized logistic regression models on longitudinal clinical data to detect the risk of nonfatal suicide attempts in adolescents. Tsui et al [20] leveraged NLP models on clinical notes along with 4 ML techniques, including Naïve Bayes, LASSO regression, random forest, and an ensemble of extreme gradient boosting, for the prediction of first-time suicide attempts. Su et al [21] used LASSO logistic regression to predict suicidal behavior among children and adolescents based on their longitudinal clinical records, identifying both short- and long-term risk factors. Barak-Corren et al [22] developed Bayesian models using a retrospective cohort approach to predict future documented suicidal behavior.

Previous studies used traditional positive-negative ML classifiers to identify/predict instances of self-harm in health records. However, due to undercoding common in mental health data, unlabeled instances contain both positive (diagnosed and undiagnosed) and negative (unaffected) cases, leading to biased classification and predictions. To address this, we applied a novel positive and unlabeled (PU) learning algorithm, PULSNAR (positive unlabeled learning selected not at random) [24], to Veterans’ EHR data to estimate the proportion of Veterans with ever self-harm. Notably, we are not predicting future self-harm, but rather classifying whether patients had experienced self-harm at any point during the study period. PU learning is a semisupervised approach that uses labeled (coded) positive examples and unlabeled (uncoded) examples containing an unknown mixture of positives and negatives [24].

To our knowledge, this is the first study to use PU learning algorithms to estimate the proportion of Veterans ever with self-harm imputing uncoded individuals in Veterans’ EHR data. By applying PULSNAR, we aim to improve the detection and estimation of self-harm prevalence among US Veterans, demonstrating a framework applicable to detecting other undetected mental health diagnoses. This approach supports earlier screening as well as novel intervention strategies to reduce self-harm and suicide rates in this high-risk population. Our findings are likely to improve awareness of risk factors of self-destructive behaviors among Veterans and highlight the broader use of PU learning in mental health informatics.

## Methods

### PU Learning Background

In many real-world applications, annotating all records can be challenging, expensive, or even impossible due to the volume of data. Often, only positive instances are labeled, leaving a considerable portion of the data unlabeled [25]. Notably, an unlabeled instance does not necessarily indicate a negative case because the absence of a diagnosis code does not confirm the absence of a condition. Given that only a fraction of records are labeled, learning from PU data has emerged as an active area of research [24-32]. The majority of current PU learning methods are based on the “selected completely at random” (SCAR) assumption [25-30], which posits that labeled positives are independent and identically distributed (i.i.d.) random samples from the positive distribution, meaning that the probability of an instance being labeled as positive is independent of its attributes [26]. However, in real-world applications, this assumption often does not hold due to labeling bias; for example, patients with more severe or specific symptoms are more likely to be labeled with a disease than those with milder or unspecific symptoms.

Despite the prevalence of real-world non-SCAR PU data, only a few studies have focused on PU learning under the non-SCAR assumption [24,31,32]. In this study, we have applied PULSNAR, a PU algorithm based on the SNAR (selected not at random) assumption. PULSNAR uses a divide-and-conquer approach to cluster SNAR positives into subtypes and estimate the proportion of each subtype among the unlabeled using the PULSCAR (positive unlabeled learning selected completely at random) algorithm. Under the SNAR assumption, the probability that a positive example is labeled depends on its attributes [24,32], which is more appropriate for health care data for which labeling bias is prevalent.

### PU Learning Algorithms to Estimate the Proportion of Self-Harm Among Uncoded Individuals

We found that existing state-of-the-art PU learning methods suffer from scalability issues and fail to execute on large datasets [24]. As a result, in earlier work, we developed 2 novel PU learning algorithms aimed at estimating the proportion (*α*) of positive instances among unlabeled examples and subsequently imputing these instances: PULSCAR for when the SCAR assumption holds, and PULSNAR for when it does not. What sets our methods apart from other PU methods is that they not only estimate *α* but also calculate calibrated probabilities using the estimated *α*, leading to markedly improved classification performance. We focus our analysis on the application of the PULSNAR framework to universally available structured EHR data; extensive methodological comparisons demonstrating its superiority over standard supervised baselines have been detailed in our prior work [24,33].

EHR data may contain various types of self-harm events, such as hanging, poisoning, cutting, etc, and the likelihood of coding these different types of self-harm may differ due to the severity of the condition and the source and nature of the underlying trauma. Therefore, it becomes evident that the SCAR assumption may not hold for self-harm data, and SCAR-based PU methods may not be suitable for such data. Recognizing this limitation, we applied the PULSNAR algorithm to estimate the proportion of self-harm-uncoded patients in an EHR dataset of US Veterans.

**Figure 1.**
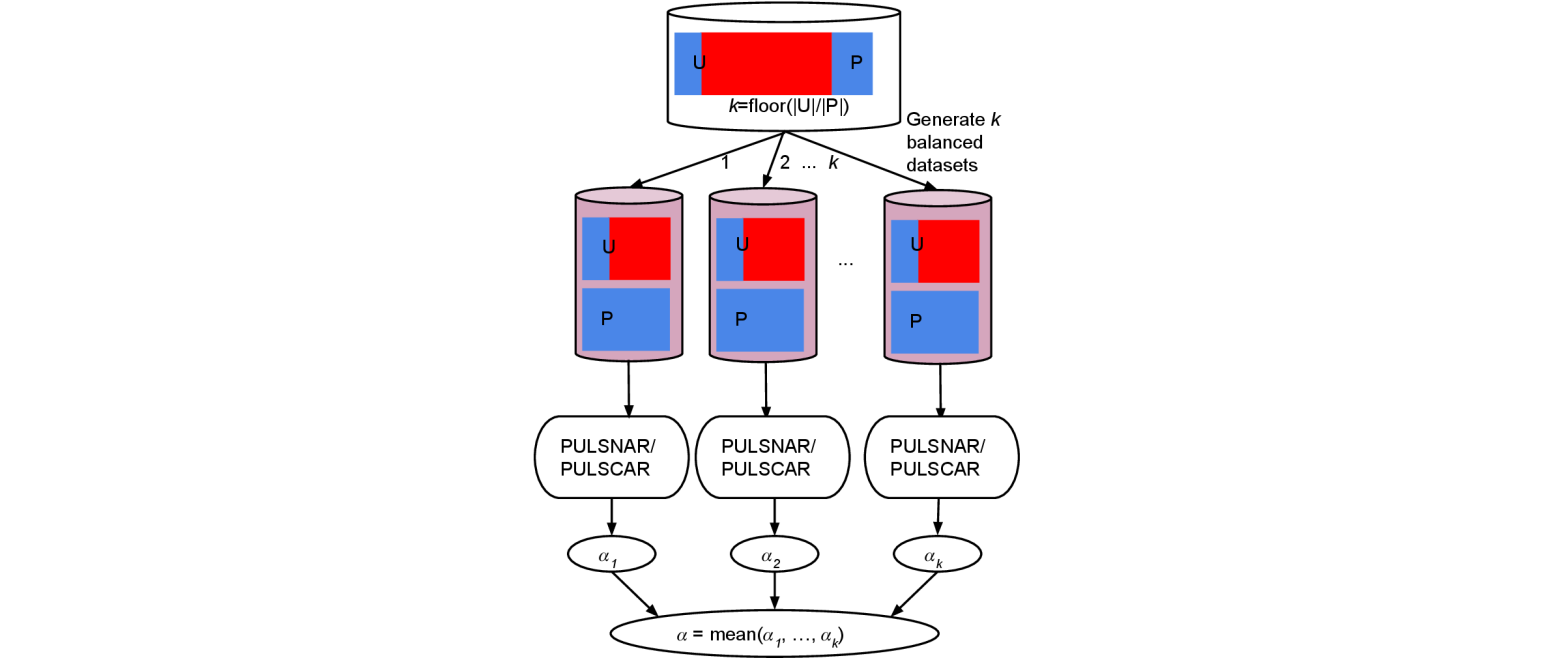
Steps for *α* estimation when the dataset has high class imbalance. The mix of blue and red represents the unlabeled set (U), and blue represents the positive set (P). To address class imbalance, *k* balanced datasets are generated, where *k*=*floor*(|unlabeled|/|labeled|). Each balanced dataset includes all labeled records and a randomly selected subset of unlabeled records of comparable size. The PU algorithm is applied to each of the *k* balanced datasets to estimate the proportion (*α*) of positives within the unlabeled set. The final *α* estimate is obtained by averaging the *k* individual *α* estimates. PULSCAR: positive unlabeled learning selected completely at random; PULSNAR: positive unlabeled learning selected not at random.

When dealing with highly class-imbalanced training datasets, ML algorithms often exhibit a bias toward the majority class, struggling to effectively generalize patterns from the minority class, which yields biased predictions [34]. Since only a tiny fraction of records were coded for self-harm in EHR data, we adopted a random undersampling approach [35], where we transformed the imbalanced dataset into *k* balanced datasets (as illustrated in Figure 1), where *k=floor*(|unlabeled|/|labeled|). Each balanced dataset comprised all labeled self-harm records along with a similar number of unlabeled records. Subsequently, we applied PULSCAR and PULSNAR algorithms (with XGBoost as the classifier) to each balanced dataset to estimate the proportion (*α*) of self-harm among the unlabeled records. The final *α* was determined by computing the mean of the *k* estimates of *α*. We performed hyperparameter tuning to determine optimal values for the XGBoost parameters. To better explore the high-dimensional covariate space, we used aggressive feature subsampling combined with a low learning rate and a large ensemble of deeper trees. This configuration limits early dominance by a small set of predictors and promotes broader exploration of the covariate space. With this sampling scheme, deeper trees provide more opportunities for additional variables to enter each tree and enable the model to capture complex interactions among covariates. Although deeper trees may increase the risk of overfitting, the large sample size and cross-validation procedures mitigate this risk. The final XGBoost parameter values were *max_depth*=12, *n_estimators*=400, *learning_rate*=0.05, *min_child_weight*=1, *colsample_bytree*=0.3, *colsample_bylevel*=0.6, *n_jobs*=32, *random_state*=0, and *objective=‘binary:logistic’*. Other parameters were kept at their default values.

### Data Source

For this study, we used the Veterans Health Administration (VHA) EHR data (from October 1, 1999, to August 31, 2019) available in the OMOP CDMv5 (Observational Medical Outcomes Partnership Common Data Model) [36] format, selecting a random 25% sample of 1,329,120 Veterans (out of 5,316,480). The only inclusion criterion was that Veterans had at least 2 years of enrollment. Ethical approvals and data use agreements were obtained from the appropriate institutional review boards to ensure compliance with privacy and confidentiality regulations.

### Phenotyping and Covariate Selection

A self-harm phenotype was defined by the presence of one or more *ICD-10-CM* (*International Classification of Diseases, 10th Revision, Clinical Modification*) or *ICD-9-CM* codes (Table S1 in Multimedia Appendix 1). These codes encompass all instances of intentional self-harm or suicide attempts by any means, including a history of self-harm. Patients with any of these codes were labeled as positive cases, while all others remained unlabeled. Accurate identification of self-harm events in structured data is complicated by self-directed violence nomenclature and coding rules that require documentation of clinical intent. Injury and poisoning codes must distinguish accidental events, intentional self-harm, assault, or undetermined causes, and in the absence of clear documentation, intent is often coded as accidental. These challenges are not unique to VHA; they affect coding practices in non-VHA systems and research cohorts as well.

**Figure 2.**
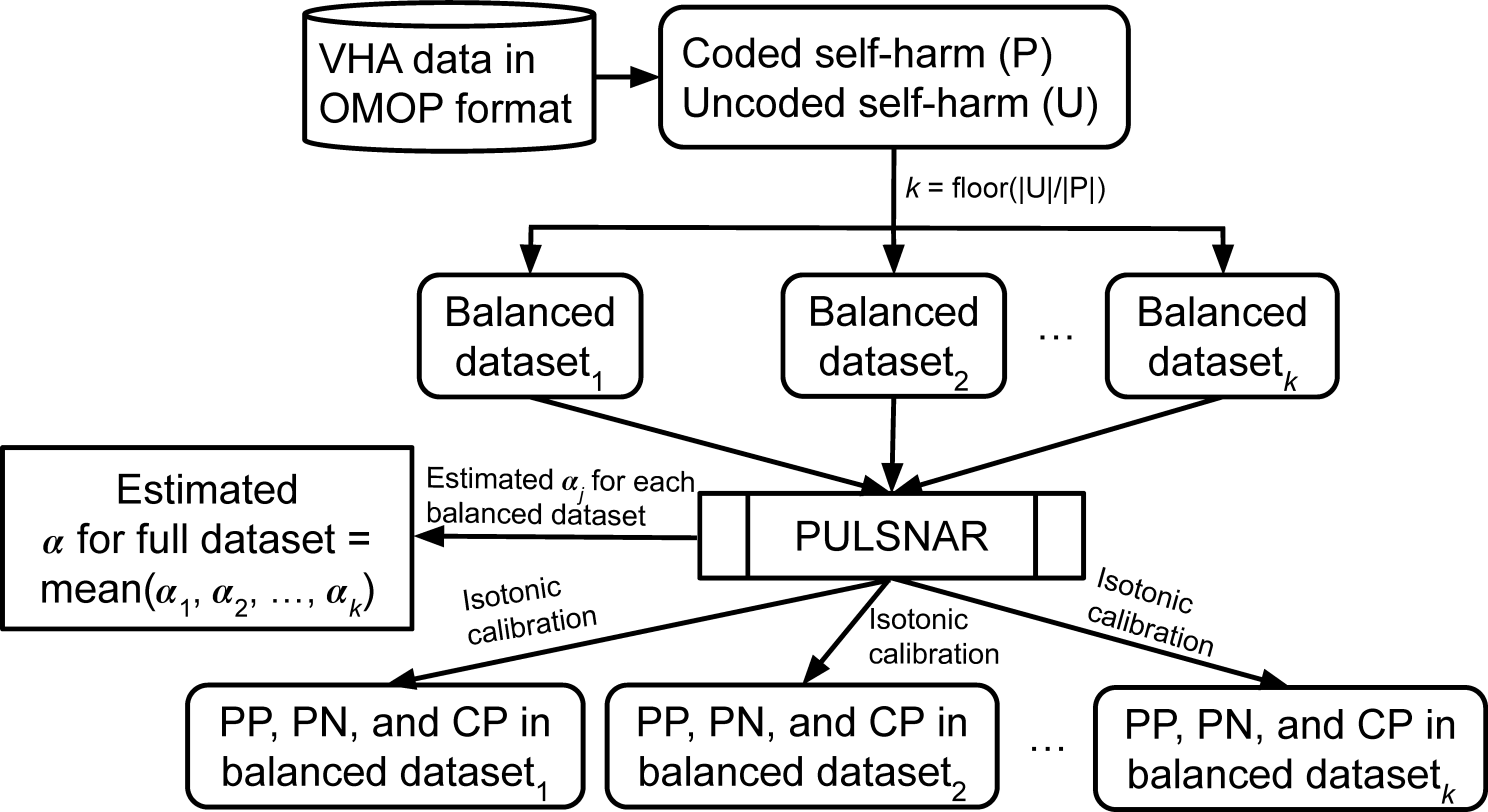
Study schema. First, *k* balanced datasets were generated, and the PU learning method was applied to each of them. The parameter *α_j_* was estimated for each balanced dataset, and the final *α* for the complete dataset was computed by taking the mean of the *k* estimated values *α*_1_*,...,α_k_*. Using the estimated *α*, the PU method calculated the calibrated probability of being labeled as positive for each uncoded individual. These calibrated classifications were then used to determine probable positive (PP) and probable negative (PN) individuals among the uncoded individuals. CP: coded positive; OMOP: Observational Medical Outcomes Partnership; PULSNAR: positive unlabeled learning selected not at random; VHA: Veterans Health Administration.

Covariates included patient age at enrollment and the presence/absence of medical conditions, procedures, and clinical observations over the duration of patient observation. *ICD-9-CM* and *ICD-10-CM* diagnosis codes were mapped to their Systematized Nomenclature of Medicine (SNOMED) equivalents (and all ancestors thereof) using the OMOP vocabulary [37]. Procedure codes from *ICD-9-CM Volume 3* (*ICD-9-CM V3*), the *ICD-10 Procedure Coding System* (*ICD-10-PCS*), and *Current Procedural Terminology, Fourth Edition*, were mapped to *ICD-10-PCS* concepts (and all ancestors thereof). Overall, from a dataset of 1,329,120 Veterans, we selected 159,049 covariates for use in the PU learning algorithm. Codes used to define the self-harm phenotype (Table S1 in Multimedia Appendix 1) were excluded from the covariate list to prevent data leakage. Since each patient had an average of only 1203 nonzero features, a compressed sparse row (CSR) matrix with 1,329,120 rows (patients) and 159,049 columns (covariates) was created as input for the PU models. Covariates were encoded as binary values (0/1) in the CSR matrix; if a covariate was not present in an individual’s data, it was set to 0, and if it was present, it was set to 1. Therefore, no covariate included in the modeling framework contained missing values. As a result, tests for missingness (eg, missing completely at random testing) and multiple imputation procedures were not applicable for our study. Figure 2 illustrates the complete schema of our study.

### Chart Review Process

To validate our model’s classifications, we conducted a chart review of a random sample of unlabeled individuals whose calibrated probabilities fell into each 1% probability bin. Due to the absence of unlabeled individuals in the calibrated probability bins in the first and last two percentiles, the selection was limited to 97 individuals. To ensure rigorous clinical validity, a comprehensive set of search keywords was established a priori in direct collaboration with our clinical psychiatrist coauthor (GV). Four informaticists (coauthors CGB, DvdG, JJY, and CGL) used these clinically vetted keywords and search utilities to identify and extract potential evidence of suicidal or nonsuicidal self-harm from combined charts, which often exceeded 500,000 lines per patient. Interrater reliability was assessed at this stage to ensure consistency among the informatics reviewers. Crucially, the extracted clinical evidence for each of the 97 cases was subsequently evaluated and discussed in detail with the psychiatrist. Rather than limiting clinical oversight to disagreement resolution among informaticists, this joint case-by-case review ensured that definitional and semantic complexities (eg, distinguishing when chronic substance abuse strictly constitutes intentional self-harm) were uniformly adjudicated by a mental health clinician. Through this expert-guided process, individuals were manually classified as positive (class 1) or negative (class 0) for ever self-harm. Finally, we compared the sum of the PULSNAR-calibrated probabilities of the 97 individuals with the sum of their consensus labels identified through expert chart review.

### Calibrated Self-Harm Prevalence Estimates for Veterans

To calibrate the PULSNAR-estimated fraction of patients with self-harm against consensus chart review results, we applied a bias-only logit shift—a logistic transformation that adds a uniform constant offset (*c*) to the logits of the original probabilities without altering their ordering. Mathematically, for each probability *p_i_,* the logit is computed as $l_{i}=\log\left(\frac{p_{i}}{1-p_{i}}\right)$; *c* is solved numerically (via root-finding) such that $\sum_{i=0}^{|U|}\frac{1}{1+\exp\left(-(l_{i}+c)\right)}$ is equal to the number of positives confirmed by chart review. The adjusted probabilities are ${\hat{p}}_{i}=\frac{1}{1+\exp\left(-(l_{i}+\hat{c})\right)}$. This method preserves relative ordering and can shift probabilities lower when correcting overestimation, providing exact sum alignment to the gold standard. We quantified calibration uncertainty using a 100,000-sample bootstrap, sampling chart-reviewed observations with replacement, solving for *c* each time to produce an empirical distribution. The 2.5th and 97.5th percentiles formed the 95% CI for *$\hat{c}$*, which we then applied to the unlabeled population to bound the total estimated positives.

### PULSNAR Classification vs Expert Chart Review

The probability of a self-harm diagnosis was stratified into low (probability < 25%), intermediate (25% ≤ probability ≤ 75%), or high (probability > 75%) categories. These stratified probability categories (which also serve as a proxy measure for the underlying behavioral risk or detection and identification of such) were compared with the outcome of the chart reviews stratified into unanimous decision against self-harm (ie, all reviewers agreed that no evidence of self-harm was found in the patient chart), dissenting evidence for self-harm (ie, some but not all reviewers agreed on self-harm), and unanimous decision for self-harm (ie, all reviewers agreed on self-harm).

### Ethical Considerations

Ethical approval for this study was obtained from the institutional review board and research and development committees of the VA New Mexico Healthcare System (H2947), University of New Mexico Health Sciences Center (20-477), and VA Tennessee Valley Healthcare System (#1576576). The requirement for informed consent was waived because the study involved secondary analysis of existing electronic health record data and posed minimal risk to participants. All data were deidentified prior to analysis, and access to the data was restricted to authorized study personnel and maintained on secure, password-protected VA servers to ensure privacy and confidentiality. No participants were contacted for this study, and no compensation was provided. No identifiable individual-level information or images are included in this manuscript or supplementary materials.

## Results

### Patient Characteristics

Applying our inclusion and exclusion criteria, we identified 1,329,120 individuals (1,193,563 males and 135,557 females) for the study. Table 1 summarizes the key demographic and clinical characteristics of the study population.

**Table 1.** Demographic and clinical characteristics of patients with and without coded self-harm selected from Veterans Health Administration electronic health record data (October 1, 1999, to August 31, 2019). The listed comorbidities represent key covariates identified as important features by the XGBoost^a^ model within the PULSNAR^b^ algorithm.

Patient characteristics (n=1,329,120)	Coded for self-harm (n=24,625, 1.85%)		Uncoded for self-harm (n=1,304,495, 98.15%)		OR^c^ (95% CI)
	Patients, n (%)	95% CI	Patients, n (%)	95% CI	
Male	20,723 (84.15)	83.69‐84.61	1,172,840 (89.91)	89.86‐89.96	0.60 (0.58‐0.62)
Female	3902 (15.85)	15.39‐16.31	131,655 (10.09)	10.04‐10.14	1.68 (1.62‐1.74)
Age (years), mean (SD)	38.39 (12.17)	—^d^	48.76 (15.04)	—	—
Age (years)					
0‐19	1301 (5.28)	5.01‐5.57	32,128 (2.46)	2.44‐2.49	2.21 (2.09‐2.34)
20‐29	6126 (24.88)	24.34‐25.42	168,060 (12.88)	12.83‐12.94	2.24 (2.17‐2.31)
30‐39	5424 (22.03)	21.51‐22.55	164,091 (12.58)	12.52‐12.64	1.96 (1.90‐2.02)
40‐49	7261 (29.49)	28.92‐30.06	257,077 (19.71)	19.64‐19.78	1.70 (1.66‐1.75)
50‐59	3712 (15.07)	14.63‐15.53	365,610 (28.03)	27.95‐28.10	0.46 (0.44‐0.47)
≥60	801 (3.25)	3.03‐3.48	317,529 (24.34)	24.27‐24.41	0.10 (0.10‐0.11)
Comorbidities					
Personality disorder	2536 (10.30)	9.92‐10.68	5627 (0.43)	0.42‐0.44	26.48 (25.22‐27.82)
Bipolar disorder	2374 (9.64)	9.27‐10.02	5887 (0.45)	0.44‐0.46	23.53 (22.38‐24.72)
Schizophrenia	944 (3.83)	3.60‐4.08	2277 (0.17)	0.17‐0.18	22.79 (21.09‐24.64)
Major depressive disorder	4412 (17.92)	17.44‐18.40	13,572 (1.04)	1.02‐1.06	20.76 (20.00‐21.53)
Psychoactive substance use disorder	3783 (15.36)	14.91‐15.82	10,933 (0.84)	0.82‐0.85	21.47 (20.63‐22.34)
Posttraumatic stress disorder	3563 (14.47)	14.03‐14.91	10,369 (0.79)	0.78‐0.81	21.10 (20.27‐22.00)
Attention-deficit/hyperactivity disorder	509 (2.07)	1.89‐2.25	1266 (0.10)	0.09‐0.10	21.72 (19.56‐24.11)
Chronic pain	2538 (10.31)	9.93‐10.69	6977 (0.53)	0.52‐0.55	21.37 (20.37‐22.41)
Adjustment disorder	2748 (11.16)	10.77‐11.56	7763 (0.60)	0.58‐0.61	20.98 (20.03‐21.97)
Sleep disorder	3570 (14.50)	14.06‐14.94	10,744 (0.82)	0.81‐0.84	20.41 (19.59‐21.25)
Traumatic brain injury	56 (0.23)	0.17‐0.30	101 (0.01)	0.01‐0.01	29.45 (20.84‐41.21)
Anxiety disorder	4424 (17.97)	17.49‐18.45	13,855 (1.06)	1.04‐1.08	20.40 (19.66‐21.16)
Induced psychotic disorder	21 (0.09)	0.05‐0.13	34 (0.00)	0.00‐0.00	32.75 (18.05‐58.10)

a XGBoost: Extreme Gradient Boosting.

b PULSNAR: positive unlabeled learning selected not at random.

c OR: odds ratio.

d Not applicable.

### Performance of PU Models

Table 2 presents the estimated proportion of self-harm cases by the PULSCAR and PULSNAR algorithms. The proportion of individuals coded for self-harm was only 1.85% (24,625/1,329,120). As expected, PULSCAR provided a lower *α* estimate (21,524/1,304,495, 1.65%) compared to PULSNAR (114,404/1,304,495, 8.77%), because the EHR self-harm data of Veterans did not satisfy the SCAR assumption. PULSCAR estimated 3.47% (46,120/1,329,120) of the individuals with coded and imputed self-harm, while PULSNAR provided a higher estimate of 10.46% (139,026/1,329,120).

**Table 2.** Performance of the PULSCAR^a^ and PULSNAR^b^ algorithms in imputing uncoded self-harm cases from Veterans Health Administration (VHA) electronic health record data. The parameter *α* denotes the estimated proportion of uncoded self-harm cases identified by each method.

	VHA (ever self-harm)
Records, n	1,329,120
Coded self-harm	24,625 (1.85%, 95% CI 1.83%‐1.88%)
Covariates, n	159,049
Covariate types	3 (condition, procedure, and observation)
Important covariates, n	1302
*α* using PULSCAR	1.65%
*α* using PULSNAR	8.77%
Coded+imputed self-harm using PULSCAR	3.47%
Coded+imputed self-harm using PULSNAR	10.46% (14 clusters)

a PULSCAR: positive unlabeled learning selected completely at random.

b PULSNAR: positive unlabeled learning selected not at random.

### Important Covariates Used by the XGBoost Model

Our covariate selection approach identified 159,049 covariates for the XGBoost model used in the PULSCAR and PULSNAR methods, but XGBoost identified only 1302 (0.82%) as informative for classification. Feature (covariate) importance was measured by the average gain (mean reduction in the loss function) contributed by each covariate. The top 15 covariates with the highest gain scores are shown in Table 3.

**Table 3.** Top 15 covariates identified by the XGBoost^a^ model, along with their corresponding gain scores. Of the 159,049 covariates included in the positive and unlabeled models based on XGBoost, only 1302 (0.82%) were identified by the model as contributing to training in our cohort. Feature (covariate) importance was quantified using average gain, defined as the mean reduction in the model’s loss function attributable to splits on a given covariate across all trees; thus, higher gain values indicate a greater contribution to improving the model’s predictive performance.

OMOP^b^ concept ID	Concept name	Gain score
438028	Poisoning by a drug AND/OR a medicinal substance	21.61
4009713	Thoughts of self-harm	14.62
440270	Poisoning by antidepressants	11.18
2840354	Medical and Surgical @ Anatomical Regions, Upper Extremities @ Supplement @ Axilla, Left @ Open @ Autologous Tissue Substitute	10.68
44782421	Acute deep venous thrombosis of the upper extremity	10.50
4133169	Operative procedure on the pelvis	10.44
434626	Borderline personality disorder	9.87
4306645	Finding of thought content	9.73
2887059	Medical and Surgical @ Gastrointestinal System @ Bypass @ Descending Colon @ Open	9.59
4181019	Cluster B personality disorder	9.49
2832323	Medical and Surgical @ Anatomical Regions, General @ Repair @ Abdominal Wall @ Percutaneous @ No Device	9.47
2841968	Imaging @ Lower Arteries @ Fluoroscopy @ Abdominal Aorta @ Low Osmolar	9.30
2762368	Supplement Thoracic Vertebra with Nonautologous Tissue Substitute, Percutaneous Approach	9.27
437456	Poisoning by an anticonvulsant	8.90
2895851	Medical and Surgical @ Upper Bones @ Excision @ Clavicle, Left @ Open	8.87

a XGBoost: Extreme Gradient Boosting.

b OMOP: Observational Medical Outcomes Partnership.

### Chart Review

Table 4 presents the interreviewer agreement among 4 reviewers across 97 uncoded self-harm cases, as well as the agreement between individual reviewers and PULSNAR, and between reviewers and the overall reviewer consensus. Table S2 in Multimedia Appendix 1 reports the pairwise Cohen κ coefficients for these agreements. Interreviewer reliability among 4 reviewers across 97 uncoded self-harm cases was substantial (Fleiss κ=0.668, *z*=16.1, *P*<.001). Notably, there were 11 patients of 39 (28.2%) where the consensus review was positive, but at least one reviewer missed the relevant evidence. Of 4×39=156 evaluations of positive charts, 17 (10.9%) were false negatives.

**Table 4.** Agreement between PULSNAR^a^ and each reviewer, between individual reviewers, between each reviewer and their consensus, and between the reviewers’ consensus and PULSNAR for 97 uncoded self-harm cases. Agreement is calculated as the percentage agreeing out of the 97 charts. Overall interreviewer reliability was substantial (Fleiss κ=0.668, *z*=16.1, *P*<.001). Among 39 consensus-positive cases, 11 (28.2%) had at least one reviewer miss relevant evidence.

	Reviewer 1	Reviewer 2	Reviewer 3	Reviewer 4	PULSNAR	Reviewer consensus
Reviewer 1	100.0%	89.7%	94.9%	82.5%	58.8%	88.7%
Reviewer 2	89.7%	100.0%	88.7%	76.3%	54.6%	82.5%
Reviewer 3	94.9%	88.7%	100.0%	79.4%	59.8%	87.6%
Reviewer 4	82.5%	76.3%	79.4%	100.0%	63.9%	85.6%
PULSNAR	58.8%	54.6%	59.8%	63.9%	100.0%	59.8%
Reviewer consensus	88.7%	82.5%	87.6%	85.6%	59.8%	100.0%

a PULSNAR: positive unlabeled learning selected not at random.

For completeness, we also calculated standard classification metrics on the 97 chart-reviewed cases using the PULSNAR probabilities versus expert consensus as the ground truth. The model demonstrated an area under the curve (AUC) of 0.6813, an *F*_1_-score of 0.5517, a precision of 0.5, and a recall of 0.6154.

### Post Hoc Calibration

After applying the bias-only logit shift on the PULSNAR probabilities for the 97 reviewed charts, using their consensus chart-reviewed self-harm labels, the sum of transformed PULSNAR probabilities equaled 39 (the expert count), with a logit shift parameter of *c*=−0.54 (bootstrap 95% CI −1.1420 to −0.0252). Applying this transformation to the 1,304,495 unlabeled patients yielded a sum of adjusted probabilities of 80,574.7, corresponding to 6.18% positives among the uncoded patients, which is lower than the PULSNAR-estimated *α* of 8.77%. This suggests that, if all notes for all Veterans without coded self-harm were chart-reviewed, 6.18% (95% CI 4.1%-8.74%) would reveal a documented history of self-harm. Thus, coded self-harm represents approximately 24,625/(80,574.7+24,625)=23.4% (95% CI 17.76%-31.51%) of all documented (coded+notes) self-harm.

### Efficiency/Scalability Quantification

To quantify the operational utility and scalability of the PULSNAR approach, we evaluated both the manual effort required for expert chart review and the computational resources consumed by our model inference across the full cohort. Expert chart review time ranged from approximately 25 minutes to 2 hours per patient chart, depending on the complexity of the record and the volume of clinical notes. Across the 97 charts reviewed in this study, this corresponds to an estimated total effort of approximately 40‐200 person-hours per reviewer. In contrast, running the PULSNAR model for inference on the entire cohort of 1.3 million records was computationally tractable, requiring approximately 63 hours of wall-clock time on a machine with 16 vCPUs, 128 GB of RAM, and 128 GB of disk space.

### PULSNAR Classification vs Expert Chart Review

Table 5 shows the comparison of PULSNAR-classified self-harm risk categories with chart review outcomes. Among those Veterans for whom expert reviewers could not find evidence of self-harm in some patient charts, PULSNAR classified the probability of self-harm as low in 35.42% of the cases and found it to be intermediate or high in the remaining 64.58% of the cases. Classifying patients as intermediate or high risk despite the absence of self-harm coding is a desirable feature, as the patient chart might also not document self-harm. Among those Veterans for whom some, but not all, expert reviewers agreed on recorded self-harm behavior in the patient chart, PULSNAR would classify the probability of self-harm to be low only in 14.81% of the cases, and intermediate to high in 85.19% of the cases. Among those Veterans for whom the expert reviewers unanimously agreed on evidence of self-harm behavior in the patient chart, PULSNAR estimated the probability of self-harming behavior as low only in 13.64% of the cases, and intermediate to high in 86.36% of the cases. Stratified as such, there was an association between expert chart reviewers and PULSNAR (Fisher exact test: *P*=.02).

**Table 5.** Comparison of PULSNAR^a^-classified self-harm risk categories (low, intermediate, and high probability) with expert chart review outcomes (unanimous against self-harm, dissenting evidence for self-harm, and unanimous for self-harm) among 97 chart-reviewed uncoded cases. There was a statistically significant association between PULSNAR risk categories and chart review outcomes (Fisher exact test, *P*=.02).

Expert review	PULSNAR classification		
	Low	Intermediate	High
Unanimous no self-harm	35.42	50.00	14.58
Dissenting self-harm	14.81	40.74	44.44
Unanimous self-harm	13.64	68.18	18.18

a PULSNAR: positive unlabeled learning selected not at random.

## Discussion

### Principal Results

In this study, we successfully applied the novel PULSNAR algorithm to a large representative cohort of US Veterans’ electronic health records to estimate the burden of self-harm beyond what is captured in structured diagnostic codes. Our primary finding is that relying solely on structured diagnostic codes dramatically underestimates the clinically documented prevalence of self-harm. By using only structured data within a PU learning framework and calibrating predictions against expert chart review of clinical notes, our approach provides a more comprehensive estimate of self-harm prevalence and highlights the extent of undercoding in VA electronic health records. These results support our central hypothesis that PU learning can recover hidden disease burden in routinely collected health care data. Critically, our results suggest that only about 1 in 4 patients with clinical notes documenting self-harm, or a history thereof, have the condition captured in structured diagnostic codes. While the discrimination metrics (AUC=0.68) appear modest, this is primarily because the model relies solely on structured data for prediction, while the validation ground truth is derived from a comprehensive review of free-text clinical notes. Furthermore, the nonrandom, uncoded test set, which lacks confirmed negatives, is optimized not for discrimination assessment, but rather prevalence calibration, which remains the study’s primary, successful objective.

Self-harm, like many sensitive mental health conditions, is frequently underreported in EHRs due to confidentiality concerns, stigma, limited help-seeking, barriers to care, and inconsistent screening and documentation practices across health care settings [38]. Additionally, self-harm is likely underdocumented in *ICD* codes in VHA records because VHA facilities receive federal funding on a per-enrolled-patient basis rather than through per-service billing, reducing incentives for exhaustive diagnostic coding [39]. It should be noted that VHA suicide risk surveillance does not rely solely on *ICD* codes. Policy and standardized templates such as SBOR are used to systematically document suicidal behaviors and overdoses [40], meaning that *ICD* undercoding does not present a complete picture of VHA’s operational monitoring of suicide risk. This underreporting hinders accurate prevalence estimation, risk identification, resource allocation, risk modeling, and intervention design [12,13,41,42]. To address these gaps, we applied PULSNAR to estimate the proportion of Veterans with both coded and uncoded self-harm and to identify likely cases at scale, serving as a case study for broader phenotyping of undercoded mental illnesses.

Veterans’ health records often contain hundreds of thousands of lines of clinical notes, making it challenging and time-consuming for clinicians to manually identify individuals at risk of self-harm, suicidality, or other mental health phenotypes through chart review [43,44]. More broadly, the self-directed violence nomenclature itself is often confusing to frontline clinicians, and the expectation of uniform, intent-based coding is difficult to meet in routine care [45]. Because injury and poisoning codes must encode intent, incomplete or ambiguous documentation pushes events toward “accidental” categories, hindering differentiation between nonsuicidal self-harm and suicidal self-directed violence [46]. This is a fundamental limitation of current surveillance approaches across health systems, and it constrains the accuracy of both administrative data and research phenotypes. Further, while one might hope that a patient’s problem list would contain a history of self-harm if it was ever noted, we found that only 22.6% (5556/24,625) of patients with coded self-harm ever had self-harm or self-harm history recorded in their VHA problem list. Given the high workload and time constraints faced by health care providers, thoroughly reviewing lengthy patient records can significantly slow the assessment process, potentially delaying risk assessment and timely intervention [47]. Another structural barrier is the absence of required, standardized training for VHA or non-VHA clinicians on self-directed violence nomenclature and the use of structured diagnostic codes for suicide risk surveillance. In practice, documentation and coding are shaped by local culture, individual comfort, and time constraints rather than uniform training, which contributes to inconsistent recognition, labeling, and coding of self-harm and suicidal behaviors across settings [48,49]. The PULSNAR method addresses this limitation by efficiently estimating the probability of self-harm through analysis of patient data, thereby assisting clinicians in prioritizing high-risk individuals for further evaluation.

Our expert chart review of 97 unlabeled Veterans yielded 3 key insights relevant to validating algorithmic phenotypes. First, probability calibration is essential and can be achieved with limited chart review. Applying post hoc calibration to the full cohort resulted in a more conservative estimate of self-harm prevalence at 7.91%, in contrast to PULSNAR’s estimate of 10.46%. Both estimates, however, are consistent with ranges reported in prior studies of Veteran populations, supporting the plausibility of our findings [50-52]. Still, here we privilege the calibrated estimate tied to observed human review.

Second, the main human-human discrepancies were both definitional and false negatives from information overload. The largest contributor to divergence was the treatment of “punching objects” (eg, walls/doors) without stated self-harm intent. One reviewer initially counted many such episodes as nonsuicidal self-injury (NSSI) (preconsensus count 45), whereas other reviewers generally did not—applying an intent or foreseeability standard. Borderline cases (eg, punching a window with tendon laceration) were debated under a reasonable foreseeability framework, absent self-harm intent. Similarly, statements about long-horizon self-destruction (eg, “drinking myself to death”) were generally classified as substance use disorder unless the episode reflected acute, explicit self-harm intent (eg, deliberate overdose). Under an inclusive NSSI sensitivity definition that includes “punching objects” [53,54], the consensus chart review identified 39 positives. This is 9 fewer than PULSNAR’s estimate of 48, which is expected and desirable, as PULSNAR is designed to provide an upper bound on the proportion of positives among unlabeled cases. Notably, 28.2% of positives had at least one reviewer missing chart evidence—a real concern, as physicians have limited time to comb through notes, unlike our reviewers who used systematic search tools and spent more time per chart than a typical visit duration.

Third, information asymmetries between data modalities, for example, notes versus codes, explain much of the remaining gap [55]. For example, manual reviewers who examined unstructured clinical notes identified several cases of self-harm history (typically past suicide attempts prior to Veteran enrollment) that were documented only in narrative notes but not captured in structured data fields. In contrast, PULSNAR relied exclusively on structured data and would have missed these cases. Conversely, some PULSNAR “high-risk” cases lacked explicit self-harm documentation in notes but exhibited risk constellations in coded data (injuries/poisonings, major mental illness, and substance use disorders). This pattern is expected given the information asymmetry between notes and codes, reinforcing that neither source is complete [55,56].

### Contribution

We emphasize two contributions. (1) Epidemiologic impact: After calibration, PULSNAR yields a population-scale estimate of ever self-harm that materially exceeds code-based prevalence. Notably, prior Veteran studies report ever NSSI between ~6% and 16%, including samples with rates of 14%‐16% [50-52], providing an external range against which our calibrated estimate can be interpreted. (2) Operational utility: PULSNAR enables triage of >1.3 million records, focusing human effort (and/or targeted NLP) where the marginal value of chart review is highest. This offers a blueprint for identifying other undercoded mental health diagnoses. In our experience, expert review of 97 charts required substantial person-hours, whereas model inference over the full cohort is computationally tractable; thus, even moderate accuracy can generate large efficiency gains by shrinking the manual search space.

Our findings support a hybrid workflow generalizable to other undetected conditions: (1) run PULSNAR on structured data to estimate the probability of being a positive case of self-harm (or other target phenotype) for uncoded individuals, (2) select a limited number of uncoded individuals from each probability bin of PULSNAR-estimated probabilities, (3) perform chart review for those selected uncoded individuals, and (4) apply post hoc calibration using chart-reviewed labels and PULSNAR-estimated probabilities of uncoded individuals to obtain calibrated cohort estimates with uncertainty. These steps, collectively, convert a complex validation into actionable epidemiology and a scalable detection pathway for underdocumented self-harm or other mental illnesses.

### Limitations

First, this study used a single data source, VHA EHRs, whose patient population, coding practices, and care patterns differ systematically from those of other US and international health systems. Accordingly, the generalizability of our findings to Veterans treated exclusively outside the VHA or to non-Veteran populations needs validation. Second, since the true *α* is generally unidentifiable [57], PULSNAR estimates an upper bound on *α* across different positive subtypes. Thus, the corresponding predicted probabilities may be overestimated. Third, the prevalence estimate relies on the assumption that the post hoc calibration factor derived from a small, stratified sample of 97 chart-reviewed cases accurately transfers to the entire unlabeled population of 1.3 million Veterans, a potential source of error we partially addressed with bootstrap resampling to quantify uncertainty. Finally, while we validated this method using chart review for self-harm, which is often documented in notes but uncoded, this approach cannot confirm cases absent from both notes and codes. Furthermore, other conditions may follow different recording mechanisms; for instance, posttraumatic stress disorder in the VHA is typically coded if documented due to disability benefit incentives. Consequently, extending this framework to such diagnoses may require validating against the incidence of future coded diagnoses rather than concurrent notes. Because EHR data do not contain reliable true negative labels for uncoded self-harm, conventional supervised classification metrics such as AUC-ROC, precision-recall, and *F*_1_-scores cannot be unbiasedly estimated [58]. This limitation is common in PU learning settings where only confirmed positives are available [24]. The chart-reviewed subset comprised only previously unlabeled cases and was not selected as a representative validation cohort with confirmed positives and negatives. Accordingly, model evaluation focused on prevalence estimation and agreement with expert review rather than traditional supervised classification metrics.

Finally, our findings should be interpreted in the context of broader limitations of suicide risk surveillance. VHA policy and clinical operations prioritize suicidal self-directed violence within the past 12 months and rely on policy-driven documentation tools such as the SBOR [40,59], whereas our phenotyping targets ever self-harm based primarily on *ICD* coding. Self-directed violence nomenclature and intent-based coding rules are complex [60], and there is no required, standardized training for VHA or non-VHA clinicians on their application [61]; as a result, documentation and coding of self-harm and suicidal behavior are inconsistent across settings and clinical providers. These surveillance and training constraints likely contribute to undercoding and misclassification and should be considered when interpreting our prevalence estimates and model performance.

### Future Directions

Future research could validate our PU learning algorithms in other populations and health care settings and across a broader range of mental health diagnoses. Incorporating unstructured data, such as clinical notes, through NLP techniques, may further enhance the detection of uncoded self-harm instances and develop valid and reliable scales to measure self-harm. Additionally, integrating our approach into clinical workflows could facilitate real-time identification of at-risk individuals, enabling timely intervention.

### Conclusions

Our study demonstrates the effectiveness of PU learning algorithms under the SNAR assumption in identifying uncoded instances of self-harm among US Veterans. PULSNAR can support both population-level prevalence estimation and individual-level risk stratification using structured health data, although differences between structured billing codes and clinical notes may affect concordance with manual chart reviews. Our findings reveal a significantly higher prevalence of self-harm than what is captured in diagnostic codes, emphasizing the urgent need for more accurate detection and imputation methods. Our approach offers a scalable and efficient adjunct to manual chart reviews for detecting undetected mental illness diagnoses, with the potential to enhance clinical practice, inform policy decisions, support comparative effectiveness studies with imputed phenotypes [13], improve predictive modeling of self-harm and other conditions, and ultimately contribute to reducing suicide rates among Veterans and improving mental health. We encourage the adoption of similar methods in other health care systems to address undercoding challenges, improve patient outcomes, and advance the application of ML in health care analytics.

## Supplementary material

10.2196/89071 Multimedia Appendix 1 *ICD-CM* codes and Cohen κ coefficients. *ICD-CM*: *International Classification of Diseases, Clinical Modification*.

## Abbreviations

AUC: area under the curve
CSR: compressed sparse row
ED: emergency department
EHR: electronic health record
*ICD*: *International Classification of Diseases*
LASSO: least absolute shrinkage and selection operator
ML: machine learning
NLP: natural language processing
NSSI: nonsuicidal self-injury
OMOP: Observational Medical Outcomes Partnership
PU: positive and unlabeled
PULSCAR: positive unlabeled learning selected completely at random
PULSNAR: positive unlabeled learning selected not at random
SBOR: Suicide Behavior and Overdose Report
SCAR: selected completely at random
SNAR: selected not at random
SNOMED: Systematized Nomenclature of Medicine
VHA: Veterans Health Administration
XGBoost: Extreme Gradient Boosting
