# Supplementary material for: Detecting Uncoded Self-Harm in Veterans’ Electronic Health Records Using Positive and Unlabeled Learning: Retrospective Cohort Study
Source: J Med Internet Res. 2026 Jun 4;28:e89071. doi: 10.2196/89071 (PMC13235979; doi:10.2196/89071)
Supplement: Multimedia Appendix 1 [file jmir-v28-e89071-s001.docx]

**Table S1.** ICD-9-CM terms used to define self-harm phenotype.

| ICD-9-CM terms used to define self-harm phenotype:   E95{0-9}* |
| --- |
| ICD-10-CM terms used to define self-harm phenotype:  X7{1-9}*; X8{0-3}*; T14.91*; T36.{0-8}X2*; T36.92*; T37.{0-8}X2*; T37.92*; T38.{0-7}X2*; T38.8{0,1,9}2*; T38.9{0,9}2*; T39.0{1,9}2*; T39.{1-2}X2*; T39.3{1,9}2*; T39.{4,8}X2*; T39.92*; T40.{0‑5}X2*; T40.6{0,9}2*; T40.{7,8}X2*; T40.9{0,9}2*; T41.{0,1}X2*; T41.2{0,9}2*; T41.{3,5}X2*; T41.42*; T42.{0-6}X2*; T42.72*; T42.8X2*; T43.{0,1,3,4,6,8}X2*; T43.{2,5,6}{0,9}2*; T43.92*; T43.{0,2,6}{1,2}2*; T43.6{3,4}2*; T44.{0-8}X2*; T44.9{0,9}2*; T45.{0-4}X2*; T45.5{1,2}2*; T45.6{0,1,2,9}2*; T45.{7,8}X2*; T45.92*; T46.{0-8}X2*; T46.9{0,9}2*; T47.{0-8}X2*; T47.92*; T48.{0,1}X2*; T48.2{0,9}2*; T48.{3-6}X2*; T48.9{0,9}2*; T49.{0-8}X2*; T49.92*; T50.{0-8}X2*; T50.A{1,2,9}2*; T50.B{1,9}2*; T50.Z{1,9}2*; T50.9{0,1,9}2*; T51.{0-3,8}X2*; T51.92*; T52.{0-4,8}X2*; T52.92*; T53.{0-7}X2*; T53.92*; T54.{0-3}X2*; T54.92*; T55.{0,1}X2*; T56.{0-7}X2*; T56.8{1,9,X}2*; T56.92*; T57.{0-3,8}X2*; T57.92*; T58.{0,1}2*; T58.{2,8}X2*; T58.892*; T58.92*; T59.{0-7}X2*; T59.812*; T59.892*; T59.92*; T60.{0-4,8}X2*; T60.92*; T61.{0,1}2*; T61.7{7,8}2*; T61.8X2*; T61.92*; T62.{0-2,8}X2*; T62.92*; T63.{0,3}02*; T63.{0,1,3-8}12*; T63.92*; T63.{0,1,3,4,6,8}22*; T63.{0,3,4,6,8}32*; T63.{0,4}42*; T63.452*; T63.{0,4}62*; T63.072*; T63.{0,4}82*; T63.{0,1,3,5-8}92*; T63.2X2*; T64.{0,8}2*; T65.2{1,2,9}2*; T65.{0,1,3-6}X2*; T65.8{1-3,9}2*; T65.92*; T71.1{1-3,5,6,9}2*; T71.2{2,3}2*; X71*; X72*; X73*; X74*; X75*; X76*; X77*; X78*; X79*; X80*; X81*; X82*; X83*; Z91.5  Note, analysis was performed before the 2022 conversion of Z91.5 (Personal history of self-harm) to a parent code and the introduction of Z91.51 (Personal history of suicidal behavior) and Z91.52 (Personal history of nonsuicidal self-harm). |

**Table S2.** Pairwise Cohen kappa coefficients showing agreement between PULSNAR and each reviewer, between individual reviewers, between each reviewer and their consensus, and between the reviewers’ consensus and PULSNAR for 97 uncoded self-harm cases.

|  | **Reviewer 1** | **Reviewer 2** | **Reviewer 3** | **Reviewer 4** | **PULSNAR** | **Reviewer Consensus** |
| --- | --- | --- | --- | --- | --- | --- |
| **Reviewer 1** | 1.000 | 0.754 | 0.876 | 0.640 | 0.172 | 0.755 |
| **Reviewer 2** | 0.754 | 1.000 | 0.721 | 0.511 | 0.089 | 0.618 |
| **Reviewer 3** | 0.876 | 0.721 | 1.000 | 0.574 | 0.192 | 0.729 |
| **Reviewer 4** | 0.640 | 0.511 | 0.574 | 1.000 | 0.278 | 0.707 |
| **PULSNAR** | 0.172 | 0.089 | 0.192 | 0.278 | 1.000 | 0.194 |
| **Reviewer Consensus** | 0.755 | 0.618 | 0.729 | 0.707 | 0.194 | 1.000 |
